# Diagnostic value of metagenomic next-generation sequencing in detecting *Pneumocystis jirovecii* pneumonia in HIV-infected patients

**DOI:** 10.3389/fmed.2025.1567484

**Published:** 2025-03-26

**Authors:** Jiawen He, Ying Chen, Zhuxiu Jiang, Feng Li, Mingli Zhu, Zhibo Xu, Meihua Wang, Meng Tang, Yuanting Wu, Yang Li

**Affiliations:** Hangzhou Xixi Hospital Affiliated to Zhejiang Chinese Medical University, Hangzhou, China

**Keywords:** metagenomic next-generation sequencing, HIV, *Pneumocystis jirovecii* pneumonia, bronchoalveolar lavage fluid, diagnostic performance, co-infection

## Abstract

**Introduction:**

Accurate diagnosis of *Pneumocystis jirovecii* pneumonia (PJP) in HIV patients remains challenging. This study compares metagenomic next-generation sequencing (mNGS) with PCR, GMS staining, and serum β-D-glucan (BG) assays for PJP detection and co-infection identification.

**Methods:**

BALF samples from 34 HIV-positive PJP patients and 50 non-PJP controls were analyzed. Diagnostic performance metrics (sensitivity, specificity, NPV, AUC) and co-pathogen profiles were evaluated for mNGS versus conventional methods.

**Results:**

mNGS and PCR both achieved 100% sensitivity. mNGS showed higher specificity (91.3% vs. 88%) and AUC (0.898 vs. 0.940 for PCR). Co-infections were detected in 67.6% of PJP cases by mNGS, including cytomegalovirus (41.2%), Epstein–Barr virus (29.4%), and non-tuberculous mycobacteria (14.7%). GMS and BG assays exhibited lower sensitivity (64.7% and 76.5%, respectively).

**Discussion:**

mNGS offers superior specificity, accuracy, and co-infection detection compared to traditional methods. Its high NPV (100%) supports clinical utility in ruling out PJP. While resource-intensive, mNGS is a promising first-line diagnostic tool for HIV-associated PJP, particularly in polymicrobial infection settings.

## 1 Introduction

*Pneumocystis jirovecii*, a prevalent fungal pathogen, frequently triggers opportunistic pulmonary infections among individuals with compromised immune systems (1). *Pneumocystis jirovecii* pneumonia (PJP) is one of the most prevalent opportunistic infections in patients with HIV/AIDS. Before the advent of antiretroviral therapy (ART) and PJP prophylaxis, 70–80% of AIDS patients were susceptible to PJP, which is associated with a significantly high mortality rate (2). Prompt diagnosis and immediate treatment are essential for enhancing patient prognosis. However, PJP’s clinical presentation often deviates from the typical, featuring rapid progression and complexities in diagnosis, potentially leading to treatment delays and inferior outcomes. Consequently, diagnostic tools with high sensitivity and specificity have remained a critical goal in clinical practice.

The traditional diagnosis of *Pneumocystis jirovecii* pneumonia (PJP) involves the identification of characteristic cyst forms of the pathogen in respiratory specimens following Gomori Methenamine Silver (GMS) staining under microscopy. Bronchoalveolar lavage (BAL) specimens obtained through bronchoscopy are considered the gold standard for diagnosing PJP in HIV-infected individuals. Nonetheless, the positivity rate for this test is considerably low (3), and *P. jirovecii* culture in the lab remains an extremely challenging task (4). The efficacy of GMS staining as a diagnostic tool is subject to variance due to factors like the quantity of fungi, the integrity of the specimen, and the proficiency of the technologist, which could result in an underestimation of the infection’s prevalence (5). Furthermore, sensitivity may decrease with prolonged empirical treatment. Lactate dehydrogenase (LDH), a widely distributed intracellular enzyme, is detected in serum as extracellular LDH, indicating cellular injury or death. Serum LDH levels are frequently elevated in PJP cases, making it a sensitive and cost-effective diagnostic tool. However, serum LDH alone has limited diagnostic value for PJP (6). (1 → 3)-β-D-glucan (*G* test, BG), a general fungal biomarker, is extensively used in diagnosing PJP, though it does not specifically confirm PJP with a positive outcome. As a non-invasive method, it is often used in conjunction with other tests to confirm diagnosis (7). The sensitivity in HIV-infected patients is superior to that in non-HIV patients, with specificity remaining comparable (8). *P. jirovecii* polymerase chain reaction (PCR) testing, in conjunction with BAL samples, has demonstrated a high sensitivity for diagnosing PJP (9). A negative PCR result signals the likely absence of *P. jirovecii*, yet a positive result does not discern colonization from active infection.

Metagenomic next-generation sequencing (mNGS) is a high-throughput sequencing technology that directly detects pathogen nucleic acids in clinical samples and uses bioinformatics methods to analyze these sequences for rapid and comprehensive pathogen identification. Over recent years, the application of mNGS in clinical microbiological studies has seen a significant increase (10–12). Compared to traditional diagnostic methods, mNGS demonstrates significant advantages, particularly in sensitivity and the ability to diagnose mixed infections (13). Several studies have shown that mNGS has a higher detection rate for PJP compared to conventional methods (14, 15). Although mNGS has been reported to have high sensitivity for diagnosing PJP in non-HIV-infected patients (16), no studies have specifically analyzed respiratory samples from HIV-infected patients with PJP. Moreover, both mNGS and PCR detect nucleic acids, but few studies have compared the diagnostic efficacy of mNGS and PCR for PJP.

This study aims to evaluate the diagnostic performance of mNGS and PCR in detecting *Pneumocystis pneumonia* (PJP) in HIV-infected patients using lower respiratory tract specimens. By comparing different diagnostic methods, the study seeks to identify potential strategies with superior diagnostic advantages for PJP. Furthermore, the study delves into the microbiota present in both PJP-afflicted and non-PJP patients, with the goal of delivering a comprehensive microbial profile.

## 2 Materials and methods

### 2.1 Study subjects

This study included People Living with HIV (PLWH) diagnosed with pneumonia and treated at Hangzhou Xixi Hospital between May 2022 and December 2023. Eligible patients met the following inclusion criteria: (1) AIDS patients aged 18 years or older, meeting the diagnostic criteria outlined in the “Chinese Guidelines for the Diagnosis and Treatment of HIV/AIDS (2021 edition)” (17); (2) evidence of pulmonary infection on chest CT imaging; (3) collection of bronchoalveolar lavage fluid (BALF) samples for mNGS testing. The clinical diagnosis of *Pneumocystis jirovecii* pneumonia (PJP) was established through a multi-disciplinary consensus involving two senior pulmonologists and radiologists, based on clinical symptoms, laboratory tests, chest imaging, microbiological results, and treatment response. Participants were classified into PJP (*Pneumocystis jirovecii* pneumonia) and non-PJP groups according to their definitive diagnosis. The exclusion criteria were as follows: (1) age ≤18 years; (2) absence of mNGS testing on BALF samples; (3) incomplete medical records. This study was approved by the Institutional Ethics Committee, and all participants provided written informed consent.

### 2.2 Methods

#### 2.2.1 Collection of bronchoalveolar lavage fluid

BALF was collected by an experienced bronchoscopist under local anesthesia with lidocaine, following standard procedures. The sampling site was selected based on chest CT images. A total of 60 mL of sterile normal saline (3 × 20 mL) was instilled into the target subsegmental bronchus. The BALF was gently aspirated using a syringe and placed into a sterile container. The sample was immediately sent for mNGS testing and Gomori Methenamine Silver (GMS) analysis. The first 20 mL was discarded to avoid contamination, with the remaining sample collected for analysis.

#### 2.2.2 Processing of BALF samples for mNGS and DNA extraction

DNA extracted from lower respiratory tract specimens of all patients was subjected to mNGS analysis. RNA sequencing was additionally conducted for cases with suspected viral pneumonia. The samples were sent to BGI Genomics and Adicon Clinical Laboratories for sequencing, using the Illumina platform. DNA extraction was carried out using the QIAamp UCP Pathogen DNA Kit, with human DNA removed by Benzonase and Tween 20 treatment. Total RNA extraction was performed using the QIAamp Viral RNA Kit, with ribosomal RNA removed using the Ribo-Zero rRNA Removal Kit. cDNA synthesis was carried out using Thermo Fisher reverse transcriptase and dNTPs. Subsequently, nucleic acid extraction, library construction, high-throughput sequencing, bioinformatics analysis, data presentation, and pathogen data interpretation were undertaken.

#### 2.2.3 Real-time fluorescence quantitative PCR detection

After centrifugation of BALF samples at 3,500 rpm (with a centrifuge radius of 17 cm) for 10 min, the pellet was collected for DNA extraction using a fungal DNA extraction kit (Hangzhou BoRi). Real-time quantitative PCR was performed using a *Pneumocystis jirovecii* probe-based fluorescence quantitative PCR kit (Borsen). The PCR reaction mixture consisted of 35 μL of nucleic acid amplification reaction buffer, 5 μL of primer-probe mix, and 10 μL of extracted DNA. The PCR program included: a pre-incubation at 50°C for 2 min, followed by denaturation at 95°C for 5 min (1 cycle); then 40 cycles of denaturation at 95°C for 15 s, annealing/extension at 60°C for 45 s; and a final hold at 12°C for 1 min. Data analysis was performed using ABI 7500 real-time PCR software, with a cycle threshold (Ct) value of ≤37 used as the cutoff for a positive result (18).

#### 2.2.4 Gomori Methenamine Silver

Formalin-fixed samples on slides were incubated with periodic acid for 10 min, followed by staining with silver solution in a 56°C water bath for 1.5 h. The slides were then treated with 0.25% gold chloride solution, 3% sodium thiosulfate, and stained with light green. The slides were then analyzed under an immunofluorescence microscope for the final results (19).

### 2.3 Statistical analysis

Statistical analysis was performed using Stata 14.0 SE or GraphPad Prism 6.0. Continuous variables were expressed as medians with interquartile ranges (IQR) and analyzed using the Mann–Whitney *U* test. Categorical variables were delineated by absolute and relative frequencies and subjected to chi-square or Fisher’s exact tests where applicable. Sensitivity, specificity, positive predictive value (PPV), and negative predictive value (NPV) were calculated using clinical diagnosis of PJP as the reference, with 95% confidence intervals (95% CI) derived using Wilson’s method. In evaluating diagnostic performance, the area under the receiver operating characteristic (ROC) curve (AUC) was compared using non-parametric approaches. The combined use of serum β-glucan (BG) and mNGS was assessed by logistic regression, with a *p*-value of <0.05 considered statistically significant.

## 3 Results

### 3.1 Comparison of general data

Eighty-four patients were recruited for this study, comprising 34 cases of *Pneumocystis pneumonia* (PJP) and 50 cases of non-PJP pneumonia (Table 1). The median age of the PJP group was 44.2 years, while for the non-PJP group it was 47.9 years, without a statistically significant difference between the groups (*p* = 0.497). The majority of patients in both groups were male, with 88.2% in the PJP group and 84% in the non-PJP group. Comorbidities were more frequent in the non-PJP group, and 16% of these patients had received prophylactic SMZ treatment, compared to none in the PJP group. The principal symptoms experienced by PJP patients were dyspnea (64.7%), cough (58.8%), and fever (64.7%). Chest CT imaging revealed diffuse ground-glass opacities in 94.1% of PJP cases, a rate significantly higher than that observed in the non-PJP group (*p* < 0.001).

**Table 1:** Baseline characteristics of HIV patients with PJP and non-PJP pneumonia.

	PJP (*n* = 34)	Non-PJP (*n* = 50)	*p*
Baseline data			
Sex (M/F)	30/4	42/8	0.534
Age	44.18 ± 14.08	47.86 ± 16.35	0.497
Clinical symptoms			
Fever (*n*, %)	22 (64.7)	26 (52.0)	0.530
Dry cough	20 (58.8)	26 (52.0)	0.757
Dyspnea	22 (64.7)	16 (32.0)	0.029
Ground-glass opacity (GGO)	32 (94.1)	10 (20.0)	0.00
Clinical indicators			
WBC (10^9^/L)	4.09 ± 1.32	5.02 ± 2.74	0.012
Hb (g/L)	110.0 ± 25.2	113.6 ± 24.2	0.428
NE (%)	76.0 ± 8.5	69.0 ± 17.5	0.319
PLT (10^9^/L)	175.5 ± 72.3	179.9 ± 72.4	0.388
CRP (mg/L)	21.9 ± 17.1	40.8 ± 50.7	0.198
PCT (ng/mL)	0.10 (0.06–0.25)	0.07 (0.04–0.36)	0.465
PaO_2_ (mmHg)	78.1 ± 13.9	92.1 ± 19.3	0.074
CD4^+^ T cell count (/μL)	25.6 ± 18.1	190.2 ± 212.0	0.016
LDH (U/L)	339.0 ± 143.9	345.6 ± 379.8	0.560
HIV RNA (IU/mL)	335.0 (155.0–667.5)	20.8 (0.0–245.5)	0.006
ART >6 month	2 (5.9)	22 (44.0)	0.013
ART <6 month	32 (94.1)	28 (56.0)	0.013
SMZ prophylaxis	0 (0.0)	8 (16.0)	0.134
Fungal (1-3)-β-D-glucan (pg/mL)	10 (58.8)	11 (44.0)	0.040

WBC, white blood cell count (10^9^/L); Hb, hemoglobin; NE, neutrophil percentage; PLT, platelet count; CRP, C-reactive protein; PCT, procalcitonin; PaO_2_, partial pressure of oxygen; LDH, lactate dehydrogenase; ART >6 month, antiretroviral therapy >6 months; ART <6 month, antiretroviral therapy <6 months; SMZ prophylaxis, sulfamethoxazole/trimethoprim prophylaxis.

Compared to patients without PJP, those with PJP exhibited significantly elevated HIV viral loads (median 335.0 IU/mL vs. 20.8 IU/mL, *p* = 0.006) and reduced CD4 counts (25.6 ± 18.1 vs. 190.2 ± 212.0 cells/μL, *p* = 0.016). Furthermore, white blood cell counts were significantly lower in the PJP group (*p* = 0.012). While serum lactate dehydrogenase (LDH) and C-reactive protein (CRP) levels were increased in both groups, no significant disparity was found. Notably, the median *G* test titer was considerably higher among individuals in the PJP group (346.7 pg/mL, *p* < 0.05).

### 3.2 Diagnostic performance

The diagnostic performance of mNGS was compared with PCR, GMS staining, and the *G* test (Table 2). mNGS and PCR were comparable in terms of diagnostic sensitivity (*p* = 1.0), specificity (*p* = 0.774), positive predictive value (PPV) (*p* = 0.771), and negative predictive value (NPV) (*p* = 1.0). mNGS exhibited significantly greater sensitivity than GMS staining and the *G* test, highlighting its advantage in detecting low-abundance pathogens and mixed infections (*p* < 0.001). Additionally, compared to GMS staining (65.8%, *p* < 0.001) and the *G* test (59.3%, *p* = 0.003), mNGS (with an NPV of 100%) demonstrated a significantly higher NPV. However, GMS staining exhibited better specificity than mNGS (87.4%, *p* < 0.001). For patients detected with *P. jirovecii* by mNGS, 13/103 (12.6%) of the false positives were in the non-PJP group without clinical diagnostic confirmation.

**Table 2:** Comparison of the diagnostic performance of mNGS, PCR, GMS staining, serum BG, and LDH.

Method	mNGS		PCR		GMS staining		Serum BG		LDH	
	+	−	+	−	+	−	+	−	+	−
PJP	34	0	34	0	8	26	12	22	22	12
Non-PJP	8	42	6	44	0	50	18	32	22	28
Sensibility	100%		100%		23.5%		35.3%		64.7%	
95% CI, *p*-value	90.7–100		89.8–100, 1.000		10.7–41.2, <0.001		19.7–53.5, <0.001		46.5–80.3, <0.001	
Specificity	91.30%		88%		100%		64%		56%	
95% CI, *p*-value	79.2–97.6		75.7–95.5, 0.774		92.9–100, 0.006		49.2–77.1, 0.039		41.3–70.0, 0.004	
PPV	90.5%		85%		100%		40%		50%	
95% CI, *p*-value	77.4–97.3		70.2–94.3, 0.771		63.1–100, 0.324		22.7–59.4, 0.001		34.6–65.4, 0.003	
NPV	100%		100%		65.8%		59.3%		70%	
95% CI, *p*-value	91.6–100		92.0–100, 1.000		54.0–76.3, <0.001		45.0–72.4, <0.001		53.5–83.4, <0.001	

### 3.3 ROC curve analysis

**Figure 1:**
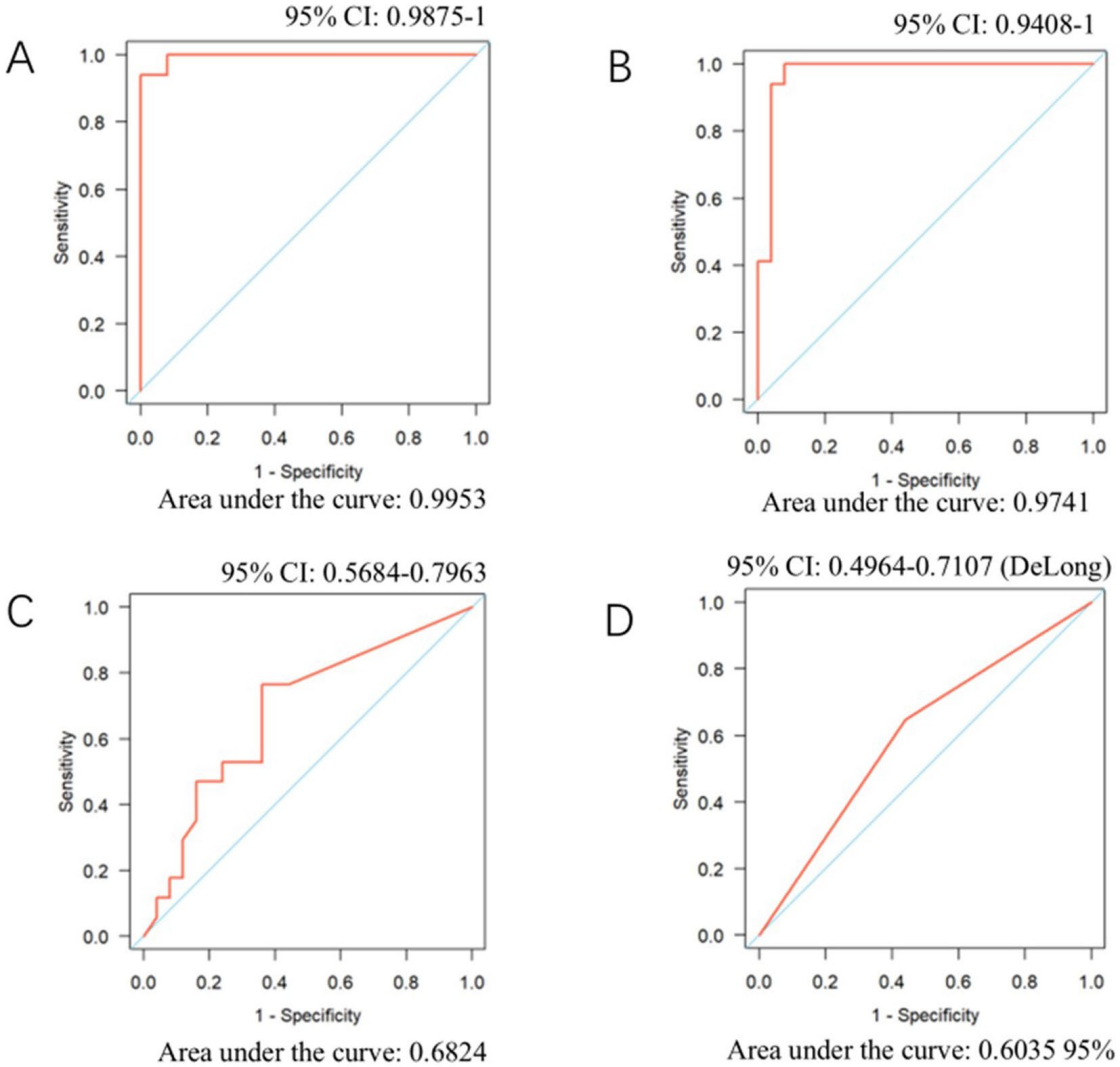
ROC curves for mNGS, PCR, serum BG, and LDH in diagnosing PJP. **(A)** NGS sequence number. **(B)** PCR. **(C)** Serum BG. **(D)** LDH.

We performed receiver operating characteristic (ROC) curve analysis on the number of sequences obtained from mNGS, the copy number detected by PCR, serum 1,3-β-D-glucan (BG) levels, GMS staining results, and lactate dehydrogenase (LDH) levels to evaluate their diagnostic performance for *Pneumocystis pneumonia* (PJP) (Figure 1). The area under the ROC curve (AUC) for mNGS was 0.9953, which was statistically significantly lower than that of PCR, whose AUC was 0.974 (*p* < 0.05). The AUC of mNGS was notably superior to that of GMS staining (AUC = 0.6035) and serum BG testing (AUC = 0.6824), with the latter two showing similar AUC values. Furthermore, we assessed the ROC curve for LDH and found an AUC value of 0.6035, which mirrored that of GMS staining.

### 3.4 Pathogen spectrum

**Figure 2:**
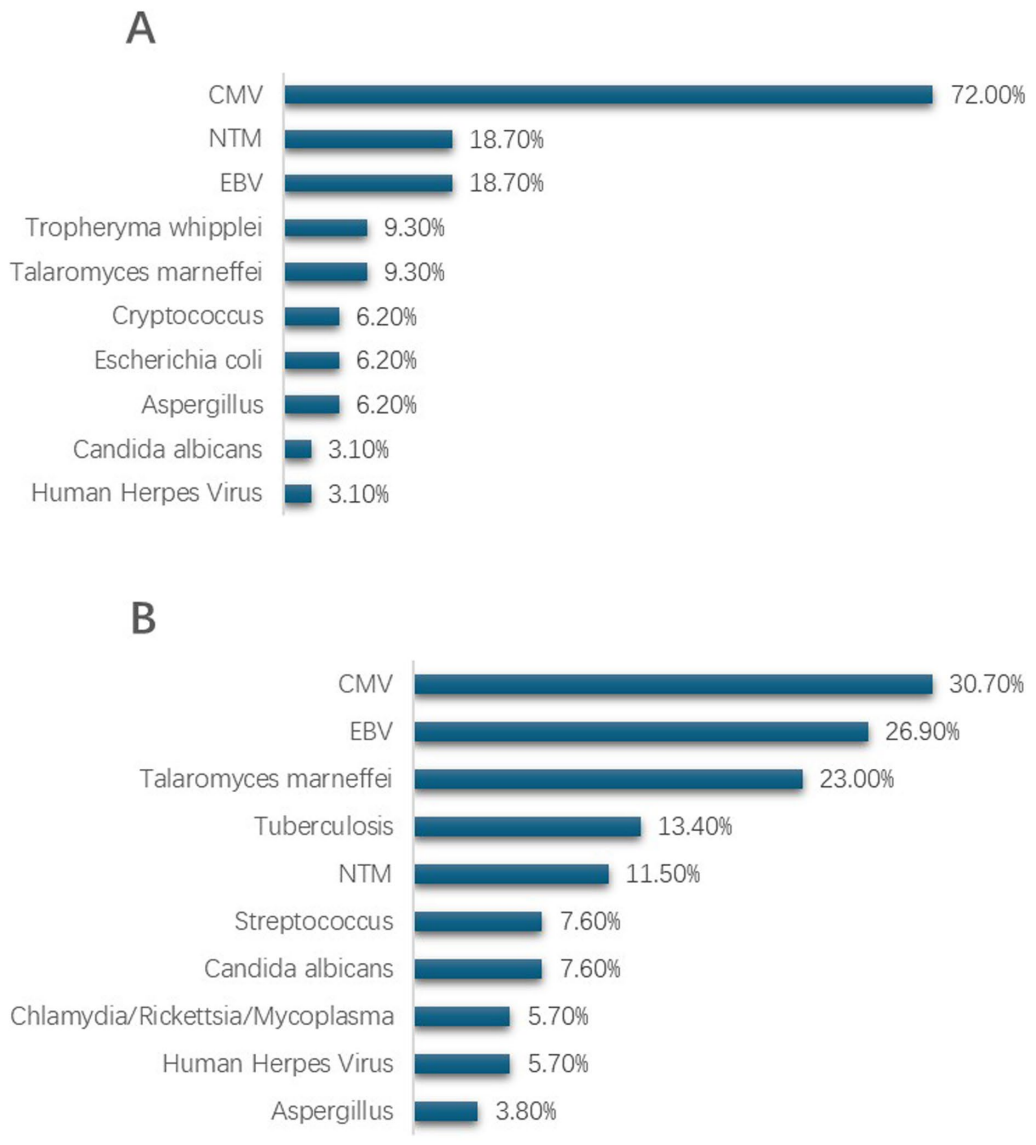
Pathogen distribution in lower respiratory tract specimens from HIV patients with PJP and non-PJP detected by mNGS. **(A)** Top 10 co-pathogens identified in HIV patients with PJP. **(B)** Top 10 co-pathogens identified in HIV patients with non-PJP pneumonia. CMV, cytomegalovirus; EBV, Epstein–Barr virus; NTM, nontuberculous mycobacteria.

In HIV patients with PJP pneumonia, co-infecting pathogens are more commonly viral. Cytomegalovirus (CMV) stands as the most frequent co-infecting pathogen in PJP patients, afflicting 72% of them, with Epstein–Barr virus (EBV) and non-tuberculous mycobacteria (18%), and Malassezia (9%) following in incidence. It is worth noting that there were no instances of tuberculosis infections detected in PJP patients through metagenomic next-generation sequencing (mNGS). Among non-PJP patients, CMV (31%) and EBV (27%) remain the most frequently identified pathogens, succeeded by Malassezia fungal infections (23%) and tuberculosis (13%) (as depicted in Figure 2).

## 4 Discussion

*Pneumocystis jirovecii* (*P. jirovecii*) pneumonia, known as PJP, is a severe opportunistic lung infection that presents a substantial risk to both HIV-infected individuals and non-HIV patients with compromised immune systems (20). Due to the lack of specificity in its clinical presentation and the inability to culture the pathogen in clinical laboratories (21), current conventional diagnostic techniques have limited sensitivity and specificity, making its diagnosis still challenging (22).

In recent years, with the development of metagenomic next-generation sequencing (mNGS), it has gained significant prominence in the diagnosis of pneumonia. Despite studies on the diagnostic effectiveness of mNGS across diverse patient cohorts and infection types (23, 24), its application in HIV-suspected opportunistic infections remains relatively under-researched.

Through examining HIV patients with PJP and those with non-PJP pneumonia, we assessed the diagnostic accuracy of metagenomic next-generation sequencing (mNGS) for detecting PJP infections in individuals with HIV and contrasted its performance with that of PCR, GMS staining, and serum β-D-glucan (BG). Our findings indicated that mNGS demonstrated a significantly greater detection rate of *P. jirovecii* in bronchoalveolar lavage fluid from HIV-positive PJP patients as opposed to serum 1-3-β-D-glucan and GMS staining, with performance on par with PCR.

The traditional diagnosis of PJP is based on the positive findings of PCR or GMS staining from lower respiratory tract specimens. Previous studies have shown that the sensitivity of GMS ranges from 31 to 97% (5). Although it is simple to perform, it highly depends on the quality of the sample and has a certain level of subjectivity. In our analysis, the sensitivity of GMS was determined to be 23.5%, paired with a specificity rate of 100%. This low sensitivity is probably attributable to the limited size of our sample cohort.

In this study, we included lactate dehydrogenase (LDH) as a diagnostic marker. LDH is an intracellular enzyme widely distributed in various tissues. Previous studies have shown that the sensitivity and specificity of serum LDH levels in the diagnosis of *Pneumocystis pneumonia* (PJP) range from 66 to 91% and 36 to 52%, respectively (25). In our study, the sensitivity of LDH (64.7%) was slightly below the reported range, while the specificity (56%) was marginally higher than previous findings. LDH levels are more likely indicative of underlying pulmonary inflammation and tissue injury rather than being pathognomonic for PJP. Nevertheless, the dynamic changes in LDH levels may still provide guidance for assessing the therapeutic response and prognosis of PJP.

In addition, we also evaluated the *G* test, which has the significant advantage of being a non-invasive diagnostic method. In this study, the specificity of the *G* test was 64%, which is close to previous studies (75%) (26), but its sensitivity was lower (35.3%). The reduced sensitivity can be linked to the increased incidence of Malassezia pneumonia among the non-PJP cohort, potentially resulting in false-positives for the *G* test. Therefore, further testing is required to validate the diagnosis.

Although both mNGS and PCR can detect *P. jirovecii* DNA, few studies have directly compared these two methods in detecting opportunistic lung infections in HIV patients. In our current PJP study, we found that mNGS and PCR showed no significant differences in diagnostic sensitivity and specificity (5). In the context of advanced-stage HIV patients experiencing immunosuppression, the prevalence of co-infections often manifests as either fungal-viral or fungal-viral-bacterial (27). While PCR can only provide results for a single pathogen, mNGS, as an unbiased detection method, can simultaneously identify multiple pathogens, giving it a clear advantage in diagnosing co-infections (28, 29).

In our study, we noted that *Pneumocystis jirovecii* pneumonia (PJP) infections in patients with HIV frequently co-occur with other pathogen infections, occurring at a high prevalence of 88.16%. Among PJP-positive cases, the most prevalent co-infections identified by mNGS were cytomegalovirus (CMV) and Epstein–Barr virus (EBV), with non-tuberculous mycobacteria (NTM) being the next most common. In non-PJP pulmonary infections, CMV, EBV, and Marneffei infection were the top three. Traditional microbiological tests (CMTs), due to their inherent limitations, often struggle to accurately identify multiple infections, especially pathogens like NTM. Our findings indicate that the co-infection of *P. jirovecii* and *CMV* is most common, a result consistent with previous studies in non-HIV infected patients (28, 29), further confirming the significant advantage of mNGS in identifying pathogens in suspected mixed pulmonary infections in HIV-infected patients. The diagnostic sensitivity for fungi, viruses, bacteria, mycobacteria, and non-tuberculous mycobacteria is notably high. This high sensitivity enables early identification of co-infections and bolsters clinical decision-making for treatment.

This study is limited by the small sample size, which may lead to potential bias in some results, such as the possibility of overestimating the sensitivity of mNGS. Future studies should increase the sample size to ensure that the results are statistically significant. Additionally, the use of BALF from different locations in the lung segments may introduce some bias, affecting the abundance of reads for PJP and, consequently, the sensitivity evaluation. While studies suggest that fewer than 14 mNGS reads could assist in diagnosing PJP colonization (19), there is no such research in the HIV-infected population, and fewer mNGS reads have been detected in BALF samples from HIV patients. It remains uncertain whether these findings indicate a transition to active PJP infection or the necessity for prophylactic sulfamethoxazole (SMZ) treatment, necessitating additional research. While PCR cannot differentiate colonization from active infection, mNGS may provide a quantitative advantage. However, standardized guidelines for HIV patients remain lacking, particularly for low-read pathogens, which warrants further investigation. mNGS is particularly recommended for PLWH with low CD4 T—cell counts, atypical radiological findings, or suspected polymicrobial infections (30). Although costly, its ability to detect co-pathogens justifies selective use. Pathogen-targeted next-generation sequencing (tNGS) offers a cost-effective alternative with comparable sensitivity, making it a practical choice for settings with limited resources. By integrating ultra-multiplex PCR with high-throughput sequencing, tNGS facilitates swift and precise identification of a broad spectrum of pathogens, even those present at low levels, and concurrently detects genes related to virulence and resistance (31–33). It is more cost-effective and may offer an advantage in detecting multiple opportunistic infections in HIV patients. Non-invasive diagnostic methods, such as nasopharyngeal swab PCR and urinary PJP antigen testing, have development potential, but their clinical application value needs further investigation.

## 5 Summary

This study underscores the superior diagnostic capabilities of metagenomic next-generation sequencing (mNGS) compared to conventional methods in identifying *Pneumocystis jirovecii* pneumonia (PJP) among HIV patients. mNGS not only provides comprehensive and accurate pathogen identification but also offers a significant advantage in detecting co-infections, enabling more precise and personalized treatment strategies.

Future studies should focus on validating these findings through larger, multicenter trials and exploring the integration of mNGS with cost-effective diagnostic approaches to improve its accessibility in clinical practice.

## Data Availability

The datasets presented in this article are not readily available because of participant confidentiality. For access, approval from our ethics committee and data custodians are required. Requests to access the datasets should be directed to the corresponding author.
